# Summary of Foreign and Domestic Medical Journals

**Published:** 1865-09

**Authors:** Frank King


### Summary.

### Summary of Foreign and Domestic Medical Journals.

PREPARED BY FRANK KING.

Dr. Gull, on the treatment of fever. (Braithwaite's Retrospect.) When the pulse in the early stage of fever, more particularly typhus and typhoid, is very frequent, the patient requires to be well supported, in order to carry him through the later stages of the disease. The rule laid down by Dr. Gull is, to keep the patient cool, sponge the skin three or four times a day, give him plenty of liquids to support the renal respiration; and plenty of fresh air to support the pulmonary respiration. The plan of giving enormous doses of stimulants is bad, if the patient is likely to die, no amount of whisky or brandy will save him, and the plan of giving him nourishment every half hour may be carried to an injurious extent, for it is very doubtful whether any of the nourishment given in severe cases is assimilated.

M. Decaisne, speaking of the effect of tobacco on the heart, (Braithwaite) says, it very frequently happens that inveterate smokers have an intermittent pulse, caused by an intermission of the action of the heart, and quite unexplainable by any organic lesion of that organ, which he terms "narcotism of the heart." The normal action of this organ will, in a large proportion of cases, be restored on the abandonment of the habit.

J. H. Bennet, M. D., on connection between phthisis and uterine disease. (Lancet, Sept. No.) The auther, who has had a large experience among consumptive women, states that the anæmia and debility caused by uterine diseases are, through their reaction on the digestive and nutritive functions, powerful predisposing causes to pulmonary consumption, especially where there pre-exists any constitutional tendency. It being a disease of defective vitality, any lesion which lowers the healthy tone of the system, such as uterine disease, leads to its development.

With the assistance of sunshine, a dry bracing atmosphere, a mild temperature, and rational sthenic treatment, hygienic, dietetic and medicinal, and the removal, if possible, of the uterine affection, Dr. Bennet has found consumption amicable to treatment. He also remarks that the treatment of chronic uterine inflammation is exceedingly tedious and harassing. The patient is below par, and for a long time does not respond to treatment. Yet it is necessary that the original disease should be treated and removed, if climate and treatment are to have a chance of success with the lungs.

Robert B. Carter, F. R. C. S., on the local application of hot and cold compresses in Ophthalmic Diseases. (London Ophthalmic Review, July No. 1865.) The application of moist, hot and cold compresses, simple or medicated, holds an important place in general surgery; and in ophthalmic surgery, such applications are of great value when discreetly used.

For cold compresses the best medium is linen, of suitable size, and from four to six layers thick, large enough to cover the orbit and immediate parts; it is necessary to provide at least three or four compresses for each eye, and to pay special attention to the following points, viz: that the compresses must not be too moist, and must be changed about every four minutes; they should be made to adapt themselves, by pressure, to all the uneven surfaces they cover. Ice itself is too severe a measure, and is said to have been followed by cataract, and opacities of the cornea.

For the application of heat, the same general method must be perused with the addition of a thermometer in the basin, to maintain the prescribed degree of temperature.

As a prominent example of the good effect to be obtained from warmth, Von Gräfe has described a disease of the cornea, usually found in children, which he calls "passive purulent infiltration," which commences as a yellow dot, occupying the centre of the cornea, and increasing quickly in size, extending deeply into the corneal tissue, and more or less superficially ulcerated. The iris becomes swollen and colored of a yellowish or redish yellow tint, but exhibits none of the results of irits. In this disease he has found great benefit from the use of warm compresses, and the instillation of atropine.

In the "loss of corneal substance," chiefly found in town-bred infants, the application of heat is extremely valuable. In corneal infiltration, resulting either spontaneously, or after operations or injuries, marked by a moderate degree of sub-conjunctival injection, with acute pain, Von Gräfe has found hot compresses of from 80° to 100° Fahrenheit a very useful remedy, and also in some cases of distinctive corneal ulcers.

In many forms of chronic keratitis and conjunctivitis, in granular lids, opaque and vascular cornea, and in diffuse opacity of the cornea without visible vessels, it is well known that the occurrence of an acute healthy inflammation is often followed by great improvement; such inflammation may often be brought about by the diligent use of heat.

The application of cold compresses will be called for in cases the opposite of those already described; cases in which it is the object of the surgeon to prevent inflammation. The chief indication for the use of cold after injuries, either surgical or accidental, is furnished by a sensation of heat in the affected eye, or by an actual elevation of its temperature. In purulent ophthalmia, of any form, the application of ice water compresses is the most important means of treatment.

Cases may arise in which medicated compresses would be preferred to the simple, such as hot infusion of opium, but they may be melted with any liquid of the proper temperature.

Henry W. Williams, M. D., on near-sightedness a disease and not an infirmity. (Boston Med. and Surg. Journal.) In this article the author records two cases to illustrate that short-sightedness, or myopia, is a disease, and not an infirmity; and that it is a consequence of an abnormal form of the globe, consisting in an elongation of its antero-posterior diameter, especially in the region surrounding the entrance of the optic nerve, where a bulging outwards of the sclerotic, termed posterior staphyloma, takes place, thereby thinning and weakening the sclerotic and choroid. The retina thus distended has its perceptive power diminished, and as the disease progresses it becomes separated, and a serous infiltration takes place between it and the choroid. As the patient advances towards middle age, cloudy flakes appear in the vitreous humor, the crystaline lens becomes opaque, which increases till cataract is formed, the removal of which, for the restoration of sight is futile.

When by the aid of the ophthalmoscope, staphyloma is found to exist to any great extent, it is unsafe to allow any employment requiring constant application of the eyes, or a stooping posture. But if only slight straphyloma exists with a healthy condition of other parts of the retina, literary pursuits may be permitted, if care is taken that the eyes be relieved by frequent intervals of rest, and the head kept, as much as possible, erect. Glasses of suitable strength, to give vision, should be worn at all times when the eyes are not employed on small objects. By so doing the eyes are spared the necessity of making efforts to recognize things at a short distance.

